# Wear Behavior between Aesthetic Restorative Materials and Bovine Tooth Enamel

**DOI:** 10.3390/ma15155234

**Published:** 2022-07-28

**Authors:** Akihiko Hatanaka, Tomofumi Sawada, Kazuyo Sen, Takahiro Saito, Kaori Sasaki, Tomoko Someya, Masayuki Hattori, Shinji Takemoto

**Affiliations:** 1 Department of Biomedical Engineering, Iwate Medical University, 1-1-1 Idaidori, Yahaba-cho, Shiwa-gun 028-3694, Iwate, Japan; info@hatanakashika.jp (A.H.); sskkaori@iwate-med.ac.jp (K.S.); takemoto@iwate-med.ac.jp (S.T.); 2 School of Dentistry, Iwate Medical University, 19-1 Uchimaru, Morioka-shi 020-8505, Iwate, Japan; d-senkaz@student.iwate-med.ac.jp (K.S.); d-saittak@student.iwate-med.ac.jp (T.S.); 3 Department of Dental Materials Science, Tokyo Dental College, 2-9-18 Kandamisaki-cho, Chiyoda-ku, Tokyo 101-0061, Japan; someyatomoko@tdc.ac.jp (T.S.); hattori@tdc.ac.jp (M.H.)

**Keywords:** two-body wear test, zirconia, lithium disilicate glass ceramic, dental porcelain, resin composite, bovine tooth enamel, wear behavior, 3Y-TZP, polycrystalline ceramics, glass matrix ceramics

## Abstract

Tooth enamel wear occurs because of daily mastication and occlusion. This study investigated the wear behavior of bovine teeth against aesthetic restorative materials in vitro. Abrader specimens were fabricated using four tooth-colored restorative materials (zirconia, lithium disilicate glass ceramic, dental porcelain, and resin composite), with bovine tooth enamel as a control. Flattened bovine tooth enamel was used as the substrate specimen. These materials were characterized by Vickers hardness tests and surface roughness measurements. Two-body wear tests between the abrader and substrate specimens were performed, and the worn topographies were evaluated using a contour-measuring instrument and 3D laser microscope. The restorative materials and bovine tooth enamel had similar surface roughness but different hardness and wear behaviors. Bovine teeth showed the largest wear in tooth–tooth contact as the abrader and substrate specimens. Compared to bovine teeth, zirconia, lithium disilicate glass ceramic, and dental porcelain showed greater hardness and less wear on their surfaces, and less substrate wear of the opposite tooth enamel. The lowest hardness resin composite showed intermediate wear on its surface, resulting in the lowest substrate wear. Accordingly, dentists should pay attention to the selection of restorative materials to reconstruct their morphologies owing to different wear behaviors.

## 1. Introduction

When biological problems such as dental caries and missing teeth in the oral cavity are present, dental restorations, including single crowns (SCs) and fixed partial dentures (FPDs), reconstruct their morphologies and oral function. In such cases, restorative material selection is a critical issue not only for dentists and dental technicians in terms of preservation of normal function and occlusal harmony, but also for patients in terms of functionality, aesthetic aspects, and cost efficiency [1,2]. Metal–ceramic restorations (MCRs), which consist of a metallic framework and veneering dental porcelain, have long been recognized as the gold standard for SCs and FPDs and still have shown similar or higher clinical success rates than all-ceramic restorations (ACRs) [3,4]. However, the use of metal-free materials for direct and indirect dental restorations, such as ceramic and resin composite, has recently increased owing to their adequate mechanical properties, aesthetic appearance, and biocompatibility. The introduction and advancement of dental computer-aided design/computer-aided manufacturing (CAD/CAM) technologies, development of tooth-colored restorative materials, and improvement of adhesive systems have made it possible for these materials to be used in clinical practice [2,5,6]. Accordingly, ACRs, such as densely sintered zirconia and reinforced glass ceramic which had less fractures or chipping of the restorations due to high fracture toughness and strength compared to dental porcelain and resin composite, showed comparable clinical outcomes to traditional MCRs [7].

Because physiological wear occurs due to repeated daily mastication and occlusion between natural teeth, the wear behavior between the tooth and the restorative material in clinical situations must be considered [8]. Ideally, restorations should have a wear behavior similar to that of tooth enamel; however, the restorative materials exhibit different properties [8,9]. These wear behaviors are influenced by various material factors (e.g.*,* mechanical and physical properties, chemical composition, microstructural and surface roughness, and homogeneity) [1,10,11,12].

Even though dental ceramics are brittle and weak and cannot withstand high tensile stresses under functional loading, polycrystalline ceramics such as zirconia can function as an alternative aesthetic restorative material; it has a wide range of applications from SCs to long-span FPDs due to its high strength and fracture toughness [6,13,14,15]. Zirconia-based materials, mainly yttria-stabilized tetragonal zirconia polycrystals (Y-TZP) and yttria partially stabilized zirconia (Y-PSZ), have been used as framework materials that are veneered with dental porcelain and/or as monolithic (full-contour) restorations [6,15]. In laboratory studies, enamel wear by polished zirconia-based materials was less than or comparable to tooth–tooth contact; however, the wear behavior was dependent on the surface topography of the restorations [16,17]. In a previous systematic review [18], it was reported that there was no significant difference in the opposite enamel wear between zirconia-based materials and enamel in vitro. Moreover, in clinical studies, enamel wear by zirconia-based materials is larger than or comparable to tooth–tooth contact [9,19,20]. Thus, these conflicting results were due to the obtained values of either or both vertical loss and volumetric loss of enamel wear by zirconia-based materials using different methods among the studies [20,21]. Furthermore, although the wear behavior of opposite zirconia-based materials has been reported in some cases [22,23], most of them are unclear because enamel wear has been the focus of previous studies [20]. Therefore, from the point of view of preserving occlusal harmony, dentists should know the wear behavior of zirconia-based materials.

Lithium disilicate glass ceramic, which consists of lithium disilicate of crystal in a glass matrix ceramic, is frequently used in ACRs [15]. Lithium disilicate glass ceramic favors the reconstruction of tooth-colored appearance as monolithic restorations when the neighboring teeth show a high translucency, but has limited indication up to short-span FPDs due to lower hardness, fracture toughness, and flexural strength compared to zirconia-based materials [2,15]. In addition, either or both vertical loss and volumetric loss between lithium disilicate glass ceramic and tooth enamel were similar, whereas zirconia-based material wear was less against enamel [10,22,24].

Dental porcelain and resin composite for indirect restorations are used as layering materials on metallic and/or ceramic frameworks for bilayered restorations because these materials are weaker and more prone to chipping and fracture than zirconia and lithium disilicate glass ceramic [12,25,26,27,28]. The wear of human enamel against MCRs was significantly higher than that against human enamel and monolithic zirconia restorations in a previous clinical study [9], whereas dental porcelain had wear behavior against human enamel similar to tooth–tooth contact and less wear than zirconia-based material in an in vitro study [10]. Moreover, the fabrication method and chemical composition of the resin composite influenced both the wear of the resin composite itself and the antagonist enamel [25,29]. The micro-hybrid resin composite containing a pre-polymerized filler and the polished zirconia-based materials showed less reduction in occlusal vertical dimension and minimized wear damage of both restorative material and tooth enamel compared to dental porcelain and other types of resin composite [26].

To date, a large number of existing studies have reported on the wear behaviors of these aesthetic restorative materials; however, the majority of these studies are unable to be compared directly because of different product materials, specimen morphologies, and testing and evaluation methods [30,31]. In particular, evidence on the wear behavior of opposite restorative materials is still a matter of debate. In addition, Chong et al. pointed out that the evidence was insufficient concerning enamel wear by these restorative materials in comparison to natural teeth [16]. Thus, limited information is available regarding the simultaneous comparison of the wear behavior of tooth enamel against these materials and natural teeth.

To overcome the dissimilarities of these different outcomes, the aim of this study was to evaluate the wear behaviors between aesthetic restorative materials and bovine tooth enamel via a two-body wear test in vitro. The null hypotheses were that (1) these restorative materials as abraders would show similar wear damage on their surfaces compared to the bovine tooth after the two-body wear tests, and (2) the wear behavior of these restorative materials on flattened bovine tooth enamel as substrates would be exhibited as well as tooth–tooth contact.

## 2. Materials and Methods

### 2.1. Materials

**Figure 1:**
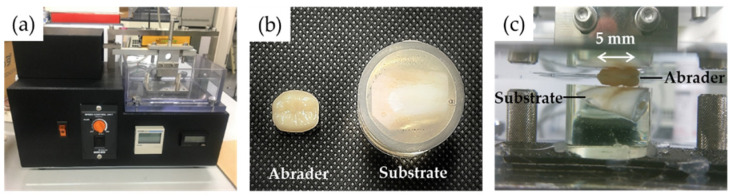
An abrasion tester (**a**), test specimens (**b**), and a two-body wear test (**c**).

A two-body wear test between the abrader and substrate specimens was performed using an abrasion tester (K236, Tokyo Giken Inc., Tokyo, Japan) (Figure 1a). Abrader specimens used four tooth-colored restorative materials and bovine teeth, and the substrate specimens were bovine teeth (Figure 1b and Table 1). Bovine teeth (mandibular incisors) were cut between the crown and root using a diamond band saw (EXAKT 30; EXAKT Advanced Technologies GmbH, Norderstedt, Germany). The abrader specimen of bovine tooth enamel (BE) was shaped, and its surface was polished so that the contact area was the same as the other groups in the test. For substrate specimens, the crown of the bovine tooth was embedded in epoxy resin (SCANDIPLEX; Fritsch Japan Co., Ltd., Yokohama, Japan). After curing, the surface of the specimen was automatically polished with 320-grit and 600-grit silicon carbide abrasive papers using a lapping machine (Doctor-Lap ML-180, Maruto Instrument Co., Ltd., Tokyo, Japan) to expose the flattened enamel surface on the bovine tooth. Bovine teeth were used in accordance with the guidelines for the care and use of laboratory animals and approved by the Institutional Ethics Committee of Iwate Medical University on 15 May 2020 (approval number: #02-004).

### 2.2. Characterization of Restorative Materials

Prior to the wear tests, the materials were characterized using a Vickers hardness test. Rectangular or disk specimens (3 mm thickness) were prepared (*n* = 3). CAD/CAM blocks of zirconia (Cercon Ht, Dentsply Sirona K.K., Tokyo, Japan) and lithium disilicate glass ceramic (e-max CAD HT; Ivoclar Vivadent K.K., Tokyo, Japan) were cut into rectangular shapes using a diamond band saw. After cutting, zirconia specimens (ZR) were sintered in a dental furnace (inFire HTC speed, Dentsply Sirona) at 1540 °C for 35 min, and lithium disilicate glass ceramic specimens (LS) were crystallized in a different dental furnace (Programat EP5010, Ivoclar Vivadent) at 850 °C for 7 min. For dental porcelain specimens (DP), porcelain enamel powder (Initial MC, GC Corp., Tokyo, Japan) was mixed with the liquid and placed into the metal jig. After removing excess moisture, the porcelain was fired at 890 °C for 1 min and glazed at 890 °C for 1 min using a furnace (Single Mat, Shofu INC., Kyoto, Japan) according to the manufacturer’s instructions. For the remaining resin composite specimens (RC), resin composite paste (Gradia Forte CT4, GC) for indirect restoration was filled to the jig, light-cured (GC Labolight LV-II, GC) for 3 min, and heat-cured at 110 °C for 15 min (Petit Oven PO-I, GC). Flattened bovine tooth enamel specimens (BE), as previously described, were used as the control group. Finally, all the test specimens were polished using 600-grit silicon carbide abrasive paper. The hardness test of each specimen was performed using a micro-Vickers hardness tester (HMV-G21, Shimadzu Corp., Kyoto, Japan) under an indentation load of 4.9 N applied for 15 s. Each specimen was measured at three different locations, and the average value was calculated.

### 2.3. Two-Body Wear Test

#### 2.3.1. Preparation of Abrader Specimens Using a Crown Model

For ZR and LS specimens, a calibration plaster model, in which abutment tooth preparation was performed on the left mandibular first molar for all-ceramic restorations, was used as a crown model. The model was then scanned using a laboratory scanner (inEos X5, Dentsply Sirona), followed by crown design using CAD software (inLab SW18.0, Dentsply Sirona) for the fabrication of all-ceramic crowns. Both the pre-sintered zirconia and pre-crystallized lithium disilicate glass ceramic blocks were milled using a CAM milling unit (inLab MC X5, Dentsply Sirona). After milling, the ZR specimens were sintered and LS specimens were crystallized, as previously described, and polished and/or glazed by clinical finishing procedures according to the manufacturer’s instructions. 

A specific mold was used to fabricate the DP and RC specimens. The mold was prepared from a ZR specimen, which imitated the occlusal surface, using an impression material (EXAFINE putty type, GC). The mold was then filled with the material. The DP specimens were then fired and self-glazed, and the RC specimens were cured as previously described and polished by clinical finishing procedures according to the manufacturer’s instructions.

Six specimens were prepared in each experimental group. For half of the abrader specimens (*n* = 3) in each group, the surface roughness (*Sa*: arithmetical mean height from the mean plane of surface) was also measured using a 3D laser microscope (LEXT OLS 4000, Olympus Corp., Tokyo, Japan) with a semiconductor laser beam of 405 nm at a range of 1280 μm × 1278 μm and calculated using software (Stream, Olympus).

#### 2.3.2. Two-Body Wear Test

The abrader and substrate specimens were fixed and placed on the top and bottom holders of the abrasion tester, respectively (Figure 1c). Prior to the wear test, the abrader specimens were observed using a digital microscope (ViTiny UM12, MicroLinks Technology, Kaohsiung, Taiwan). The contact area between the distobuccal cusp of the abrader specimen and the flattened surface of the substrate specimen was confirmed using red articulating paper. The stroke width of the abrader specimen was also verified using red articulating paper to confirm the constant movement. The wear test was performed under water immersion at room temperature using the following parameters: vertical load, 4.9 N; cycles, 30,000; frequency, 2.5 Hz; stroke width (left-to-right slide), 5 mm. 

After testing, the surfaces of both the abrader and substrate specimens were observed using a digital microscope. The worn profile in the center of the substrate specimens was recorded vertically in the direction of movement of the abrader specimen using a contour-measuring instrument (DSF600S, Kosaka Laboratory Ltd., Tokyo, Japan) to calculate the worn width and depth. The worn area and/or volume of the substrate and abrader specimens were measured using a 3D laser microscope, as previously described. In addition, the worn surfaces of the selected abrader specimens were examined using scanning electron microscopy (SEM; SU8010, Hitachi High-Tech Corp., Tokyo, Japan) at 10 kV after plasma coating with OsO_4_.

### 2.4. Statistical Analysis

Data were analyzed using software (BellCurve for Excel, Social Survey Research Information, Tokyo, Japan) at a level of significance of α = 0.05. The data were analyzed for normal distributions by the Shapiro–Wilk test and for variance equality by the Levene test. The results for Vickers hardness and surface roughness were analyzed by a one-way analysis of variance (ANOVA) followed by Tukey’s test for post hoc comparisons. As the data of wear test parameters were not normally distributed, descriptive statistics were applied using medians. These results were analyzed by a non-parametric Kruskal–Wallis test followed by Steel–Dwass post hoc test for multiple comparisons.

## 3. Results

### 3.1. Vickers Hardness

The Vickers hardness of the specimens ranged from 124 ± 4 to 1287 ± 33 (Table 2). The hardness values were significantly different among all the groups (*p* < 0.05). The ZR group exhibited the highest hardness values, followed by the LS, DP, BE, and RC groups.

### 3.2. Surface Roughness

The surface roughness (*Sa*) of the abrader specimens ranged between 6.1 ± 0.5 µm and 10.6 ± 3.6 µm (Table 3). No significant differences were observed among the groups.

### 3.3. Worn Width and Depth of the Substrate Specimens

**Figure 2:**
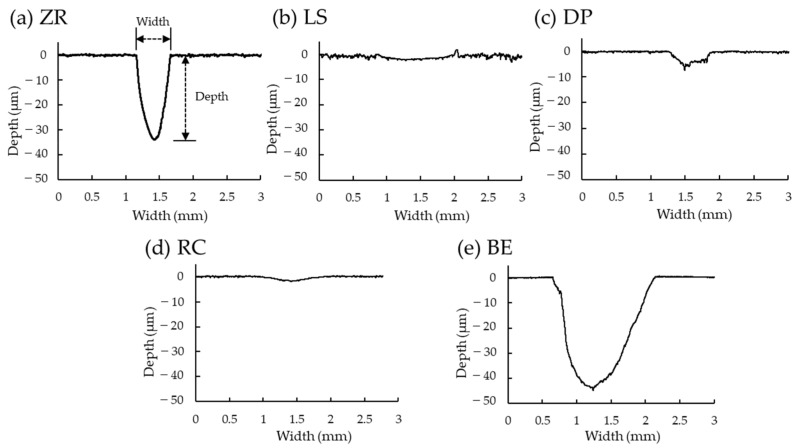
Typical cross-sectional profile of worn scratch of the substrate specimens. Abbreviations of each experimental group are shown in Table 1.

The worn width and depth of the substrate specimens and typical cross-sectional worn profiles of the specimens are presented in Table 4 and Figure 2, respectively. The BE group showed the largest worn width, which was significantly larger than that of the other groups (*p* < 0.05, Table 4). However, no significant differences were observed in the worn widths of the restorative materials (*p* > 0.05). The worn depths of the specimens were significantly different among the materials (*p* < 0.05). The ZR and BE groups showed the largest worn depths. The RC group exhibited the smallest worn depth; however, no significant difference was observed between the LS and RC groups. These differences were observed in the cross-sectional worn profiles, indicating that the BE group showed larger width and depth profiles, and the ZR group showed a larger depth profile (Figure 2).

### 3.4. Analysis of the 3D Laser Microscope Observation

#### 3.4.1. Worn Area and Volume of the Substrate Specimens

**Figure 3:**
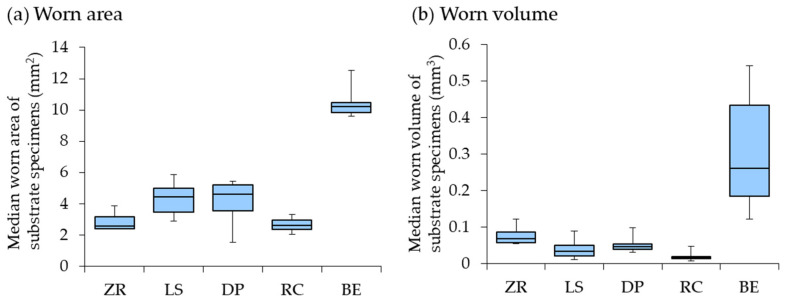
Box plots of worn area (**a**) and volume (**b**) of the substrate specimens. Abbreviations of each experimental group are shown in Table 1.

**Figure 4:**
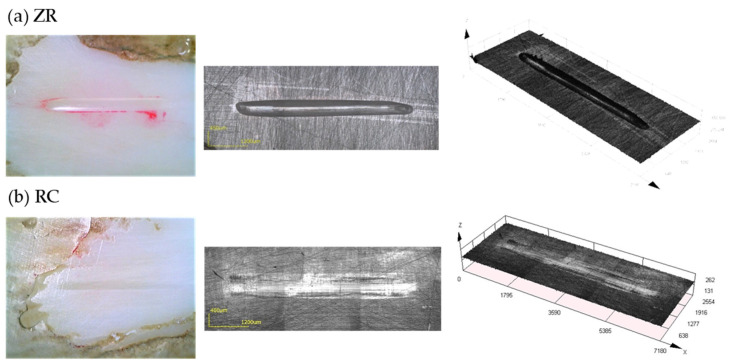
Digital microphotographs (left) and 3D laser microscope images (middle and right) of the substrate specimens after the wear test. Abbreviations of each experimental group are shown in Table 1.

The worn areas and volumes of the substrate specimens are presented in Table 5 and Figure 3, respectively. The worn area and volume of the BE group were significantly larger (Table 5, *p* < 0.05). No significant differences were observed among the restorative materials in the worn area (*p* > 0.05). The RC group showed the lowest worn volume, which was significantly lower than that of the ZR group among the restorative materials (Table 5, *p* < 0.05). In the 3D laser microscope images, the RC specimen exhibited less wear damage than the ZR specimen (Figure 4).

#### 3.4.2. Worn Area of the Abrader Specimens

**Figure 5:**
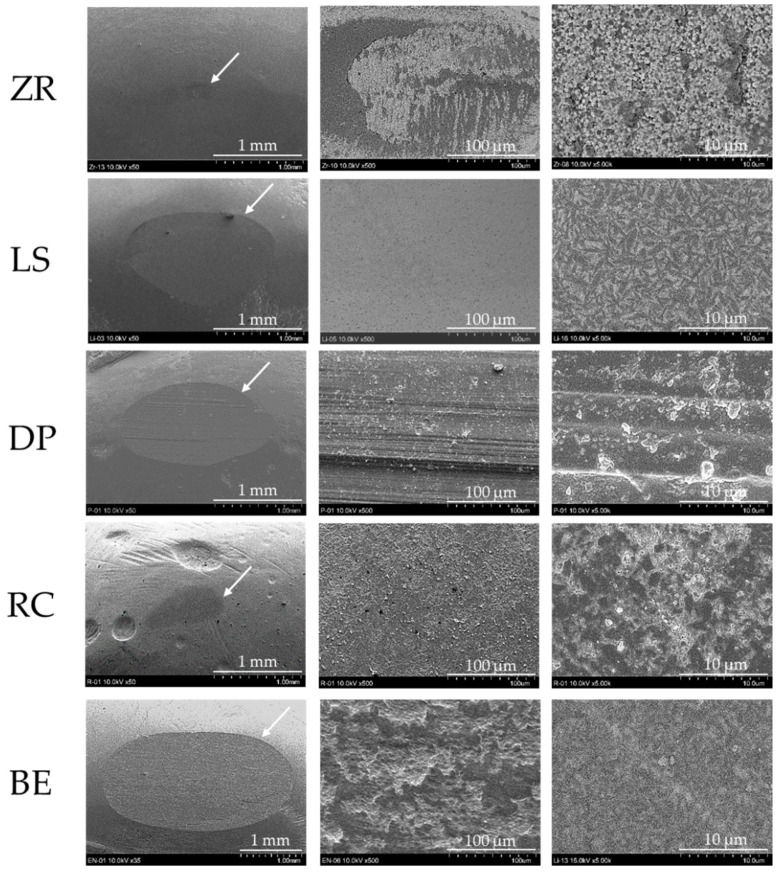
Scanning electron microscopy (SEM) micrographs (left, 50× magnification (only BE: 35× magnification); middle, 500× magnification; right, 5000× magnification) of the worn surface of the abrader specimens. White arrow indicates the wear facet area of the respective specimen. Abbreviations of each experimental group are shown in Table 1.

The worn areas and SEM images of the abrader specimens are presented in Table 6 and Figure 5, respectively. The worn area of the BE group was significantly largest (Table 6, *p* < 0.05). Among the restorative materials, the worn areas in the LS and DP groups were significantly larger than those in the RC and ZR groups (*p* < 0.05). The ZR group exhibits the lowest values. 

These differences were confirmed by microscopic observations. In the SEM images, the BE specimen showed the largest wear facet, whereas the ZR specimen exhibited the smallest wear facet (Figure 5).

## 4. Discussion

The demand for aesthetic restorative materials as metal-free restorations is increasing annually. Tooth enamel wear, which contacts the opposite natural tooth or aesthetic restorative material, leads to irreversible loss of tooth substance. However, evidence concerning the wear behavior of opposite restorative materials and their comparison with natural teeth is inadequate. In this study, the wear behavior of bovine tooth enamel against four tooth-colored restorative materials compared to bovine teeth were investigated via the two-body wear test. The results demonstrated that (1) Bovine teeth showed the largest wear area and/or volume as the abrader and substrate specimens compared to the restorative materials. (2) Dental ceramics showed less wear on their surfaces and less substrate wear of the opposite bovine tooth enamel. (3) The resin composite showed intermediate wear on its surface and the lowest substrate wear among the restorative materials. Thus, the first and second null hypotheses were rejected.

Many in vitro and in vivo studies on wear behavior against tooth enamel have been conducted. Understanding the characteristics of restorative materials is critical for the long-term clinical success of restorations in dentistry. Clinical studies are essential to characterize complex oral wear situations [32]. Unfortunately, clinical consensus is not available with regard to their wear behavior due to the large variability in individual wear behavior in patients, which accounts for about 50% of the variability [30,32,33]. It also makes clinical evaluation difficult in terms of the aspects of cost and time effectiveness. On the other hand, in laboratory studies, eight different wear testing methods of two- and/or three-body contact for dental materials have already been introduced to simulate the wear behavior by occlusal contact of antagonistic teeth in the report of International Organization for Standardization/Technical Specification (ISO/TS) 14569-2:2001 [34]. Heintze et al. pointed out that these methods are not appropriate because of the lack of description about their advantages and disadvantages in this report [30]. The wear evaluations including the simulators have different approaches owing to different operational and methodological concepts; therefore, they cannot be compared, even if efforts are made to use similar wear parameters [30]. In addition, the laboratory wear test was not strongly correlated with clinical outcomes [30,35]. Currently, there are no regulatory requirements or international standards for testing restorative materials by laboratory wear simulations [30]. However, laboratory wear tests are necessary for the consideration of materials and allow the investigation of the single parameter of wear processes and a comparative evaluation of different materials under standardized conditions, even though considerable variability should be considered in such cases [30,35,36]. Hence, in this study, a two-body wear test that simulated the attrition between the abrader and substrate specimens was used to compare the wear behavior of the materials. Attrition, which is classified as tooth wear, is defined as gradual loss by the physiological wear of tooth-to-tooth contact [37,38]. In addition, a two-body wear test is simpler than other tests such as three-body wear, abrasion, and erosion tests, because any other extrinsic or intrinsic factors can be excluded [38,39].

The crown models were fabricated using four tooth-colored restorative materials and used for the wear test in this study. The morphology of the crown model was assumed to be the same as that of the mandibular first molar in clinical practice. The distobuccal cusp of the crown model was selected and contacted the flattened surface of the substrate specimen. Kirsten et al. reported that the distribution of the maximum tensile stress was concentrated in the occlusal fissures between the mesiolingual and distobuccal cusps of Y-TZP crowns in physiological mastication behavior [40]. Moreover, the buccal cusps in the mandibular molar region act as functional cusps and are subjected to concentrated occlusal forces during chewing and biting [41]. Furthermore, the specimens were prepared using conventional manufacturing methods. CAD/CAM blocks were used for the zirconia and lithium disilicate glass ceramic specimens, whereas traditional hand-layered materials were used for the dental porcelain and resin composite specimens. These differences are dependent on the clinical indications for monolithic and bilayered restorations. Even though a variety of manufacturing methods for the respective materials exist, their consideration was excluded to focus on the comparison of the materials in this study.

**Figure 6:**
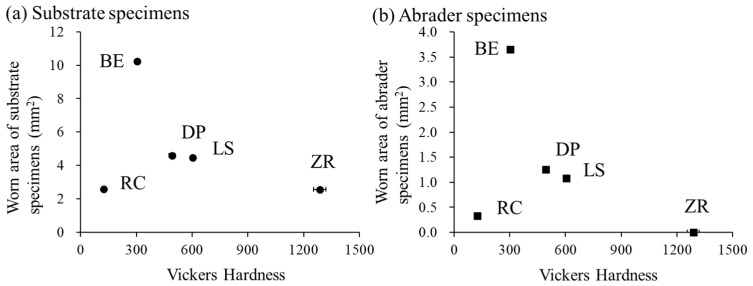
The relationships between the specimen-worn area and Vickers hardness of the abrader specimen ((**a**): worn by substrate specimens, (**b**): worn by abrader specimens). Abbreviations of each experimental group are shown in Table 1.

Dental ceramics are generally harder than human tooth enamel and metal alloys [1]. Actually, the Vickers hardness values of the three types of ceramics (ZR, LS, and DP) used here (ranging from 492 ± 16 to 1287 ± 33) were significantly larger than those of bovine tooth enamel (303 ± 14). In addition, bovine teeth have been used as abrader and substrate specimens because the Vickers hardness of human tooth enamel (approximately 274–317) is the same as that of bovine tooth enamel (approximately 300–340) [25,42,43]. These findings were consistent with previous studies [44,45,46]. The hardness differences were dependent on the microstructure of the ceramic materials including the presence or absence of the glass matrix, thereby resulting in different wear behaviors of both the abrader and substrate specimens. The specimens with greater hardness (ZR, LS, and DP) showed less substrate wear of the opposite bovine tooth enamel than the lower hardness specimens (BE) (Figure 6a). Zirconia is a well-known polycrystalline ceramic with a fine-grained crystalline structure without any glass phase, providing the largest flexural strength and fracture toughness among dental ceramics [15]. In contrast, glass ceramic (e.g.*,* lithium disilicate and feldspathic porcelain) consists of a glass matrix and crystalline phase (30–70 wt%), which are embedded in the glass matrix [47,48]. When ceramics have lower hardness, lower concentrations of crystalline phase, and smaller crystal sizes, they are more wear-friendly to tooth enamel [1]. Therefore, even though no significant differences in the worn area and volume of the substrate specimen were observed among the ceramics (ZR, LS, and DP), the worn volume of the substrate specimen in the ZR group tended to be higher than that in the LS and DP groups in this study (Table 5). These differences were also confirmed by the cross-sectional profile of worn scratches using a contour-measuring instrument (Figure 2), in which the bovine tooth wear by the ZR specimen was more lengthened in the depth direction than that by the LS and DP specimens. This finding is inconsistent with that of previous studies [10,22,49]. In these cases, the polished zirconia specimens exhibited less enamel wear than the glazed porcelain specimens. These conflicting results were due to the preparation of the DP specimens with or without glazing material during the firing steps.

Regarding the worn area among the tested ceramics, the worn area of the ZR specimens was significantly smaller than that of the LS and DP specimens (Table 6), indicating that the larger hardness material showed less wear on its surface (Figure 6b). This finding may be influenced by the hardness, microstructure, and supply method of the ceramic materials. This assumption was partially supported by previous studies [1,21,49]. In general, the wear of tooth enamel is influenced not only by the roughness and hardness of the material but also by the material surface microstructure and friction environment. In this study, the specimens were prepared using clinical finishing procedures according to the manufacturer’s instructions, and there were no significant differences in the surface roughness of the materials before the wear test (Table 2). Thus, the surface roughness was eliminated as a possible factor. The harder ZR specimen had a homogeneous microstructure without any glass phase because the homogeneous CAD/CAM blocks were milled. In contrast, glass ceramics are more inhomogeneous and not free of porosity [1,49]. For the worn area and volume of the substrate specimens, there was no difference between the LS and DP specimens (Table 5). However, the worn depth of the substrate specimen in the DP specimen was significantly deeper than that in the LS specimen (Table 4). This difference was due to some factors including the material hardness, the content of the crystalline phase in the glass ceramic, and the supply method. The LS specimens are more homogeneous by using industrially fabricated blocks with minimal flaws and contain the higher crystalline phase (70 wt%) [6,50], resulting in a shallower worn depth than the DP specimens. It has been reported that glass ceramics sustain indentation damage primarily by plastic deformation and subsequent fracture of the weaker glass matrix, whereas crystalline ceramics fail through the dislocation mechanisms of crystals in a glass matrix under indentation loads [1]. In addition, glass ceramics may provoke wear, resulting in the removal of the glass matrix and exposure of the crystalline phase, which causes surface roughening. However, Lawson et al. reported that the wear mechanism of the porcelain specimen was different from that of the zirconia and lithium disilicate glass ceramic specimens [22]. They also described that porcelain fracture during wear caused sharp asperities on its surface, resulting in abrasion opposing human tooth enamel. These differences may reflect the surface damage of the abrader specimen. These wear behaviors were also confirmed by SEM micrographs (Figure 5). The worn surfaces of the ZR and LS specimens were relatively smooth without any particles, whereas that of the DP specimen was rough owing to the presence of fractured porcelain debris on the specimen. This finding is supported by a previous study [49]. In the EPMA analysis, Hara et al. demonstrated that the components of feldspathic porcelain (Si and Al elements) were detected on the substrate (bovine enamel surface), resulting in the possibility of these particles adhering to bovine tooth enamel after the wear test, whereas the component of zirconia (Zr element) was not detected [49]. They also reported that the smooth surface of the zirconia specimen was maintained during the wear test, resulting in no detectable sign of wear loss, whereas the lithium disilicate glass ceramic specimen exhibited measurable wear on the surface owing to material experiences [22,23].

Resin composite consists of a resin matrix (20–30 wt%), filler (70–80 wt%), and a small amount of catalyst or initiator [51]. Increasing the filler content and degree of polymerization improves the mechanical properties of resin composite [25,51]. Resin composite with predominantly a combination of large and small particles of the filler, so-called hybrid types, is frequently used to increase filler content. In addition, resin composite for indirect restoration can improve the degree of polymerization through light irradiation and heating [25,28]. Therefore, the filler size, shape, and degree of polymerization of the resin composite affect tooth enamel wear [25,51]. However, it must be mentioned that the wear behaviors of tooth enamel are different between ceramics and resin composite [28]. Compared to other ceramic restorative materials, the obtained results here indicated that resin composite showed lower hardness, less tooth enamel wear, and less wear damage on its surface (Figure 6). The Vickers hardness values of the RC specimens were similar to those of a previous study that showed values over Hv 100 [25]. Suese et al. also showed that the hardness of an indirect resin composite material is higher than that of conventional light-curing resin composite materials [28]. Regarding the wear of human tooth enamel, zirconia and resin composite showed less wear than lithium disilicate glass ceramic and human tooth enamel [24]. In such a case, the difference between zirconia and the resin composite is the surface roughness. The surface roughness of the resin composite material did not change after the wear test, resulting in less tooth enamel wear [24]. This feature of the resin composite was confirmed by microscopic observations as a smooth worn surface (Figure 4 and Figure 5). These differences were owing to the wear process in which the ceramic caused a microfracture mechanism, whereas the resin composite caused adhesive wear [24]. In contrast, the wear of bovine tooth enamel did not differ among the resin composite, lithium disilicate glass ceramic, and bovine tooth enamel [52]. These differences may be dependent on the materials, testing method, and origin of tooth enamel. In summary, concerning general knowledge on two-body wear, a softer material is abraded more easily than a harder material [24]. Awada et al. reported that the difference in the elastic properties between ceramics and resin composite could be attributed to the resin content, which makes them less brittle and more flexible [53]. Therefore, the RC specimens showed smaller wear area and volume on its surface and on the bovine tooth enamel surface in this study.

Bovine tooth enamel was used not only as a substrate specimen but also as an abrader specimen because bovine tooth enamel had a thicker layer than human tooth enamel so that a large and more uniform structural area could be used. In a previous review of ten dental erosion/abrasion studies, Yassen et al. summarized that bovine tooth enamel was considered a promising substitute for human tooth enamel, even though inconsistent outcomes exist [54]. Some studies have reported no difference in hardness between human and bovine tooth enamel. The hardness of bovine tooth enamel (Table 2) is comparable to that of human enamel teeth, as previously described. In addition, the main reasons of the usage of bovine tooth are that bovine tooth enamel has a more uniform composition and is readily available, and its crystallite orientation matches that of human tooth enamel [55]. Bovine tooth enamel suffered severe wear in tooth–tooth contact as the abrader and substrate specimens in comparison to other restorative materials in this study (Table 5 and Table 6). These wear behaviors were also confirmed by SEM micrographs (Figure 5). This finding was consistent with previous studies that when tooth enamel makes contact, it causes high abrasion with visible roughness and pitting [17]. This could be explained by a previous study showing that, from the viewpoint of microstructure, the cracks were semicircular in shape and grow along the direction of the enamel rods, followed by enamel cracks that propagate along the rod sheath [56]. Yahyazadehfar et al. reported that [57] the crack growth resistance of human enamel was inhomogeneous and spatially anisotropic due to the complexity of the hierarchical microstructure and prism structure. In previous studies, tooth–tooth contact had significantly higher enamel wear than zirconia, feldspathic porcelain, and resin composite [17,24,32]. Sripetchdanond et al. explained that three-body wear occurred during a two-body wear test because the chipped hydroxyapatite particles worked as an abrasive medium [24]. In contrast, human tooth enamel showed equivalent opposing enamel wear to polished zirconia and lithium disilicate glass ceramic, whereas zirconia showed less occlusal wear than human tooth enamel and lithium disilicate glass ceramic showed equivalent enamel wear [22]. A definite conclusion of the wear mechanism is a matter of debate. Therefore, further in vitro studies are needed to clarify tooth–tooth contact. 

In this study, the measurements of the wear parameters (worn area and/or volume) of the abrader and substrate specimens using the 3D laser microscope were different (Table 5 and Table 6). This difference was due to the fact that the substrate specimens used were flattened bovine tooth enamel and simplified, whereas the abrader specimens were crown models with a more complicated cusp (edge) shape. In other words, it should be considered when compared with other studies because the obtained results would be different if the specimen morphology and testing method were changed. Moreover, it should be mentioned that the measurements of both the worn area and volume were not necessary because both variables were strongly correlated with each other [11,17].

Finally, although this study demonstrated the comparison of the wear behavior of bovine tooth enamel against tooth-colored restorative materials and natural teeth simultaneously, the variety of wear such as contact with the same material in clinical situations should be considered. In addition, it is assumed that wear behavior differs depending on the manufacturing method of the restorative materials [25,58]. Thus, further studies are needed to clarify the influence of these factors on wear behavior to establish the reliability of the findings.

## 5. Conclusions

Within the limitations of this study, the conclusions are as follows:

1. The tooth-colored restorative materials and bovine tooth enamel had similar surface roughness with different hardness, resulting in different wear behavior on their surfaces and against the opposite tooth enamel.
2. Compared to the restorative materials, bovine teeth showed the largest wear area and/or volume in tooth–tooth contact as the abrader and substrate specimens.
3. Zirconia, lithium disilicate glass ceramic, and dental porcelain showed larger hardness and less wear on their surfaces and less substrate wear of the opposite tooth enamel.
4. Although there was no difference in the wear of the substrate specimen, the worn area of the abrader specimen in zirconia was significantly smaller than that of lithium disilicate glass ceramic and dental porcelain.
5. Among the restorative materials, resin composite for indirect restorative showed the lowest hardness and intermediate wear on its surface, resulting in the lowest substrate wear.

Thus, dentists should pay attention to the selection of restorative materials to reconstruct their morphologies owing to different wear behaviors.

## Figures and Tables

**Table 1:** Materials used as abrader specimens in this study.

Material	Product	Composition *	Manufacturer	Lot No.	Code
Zirconia (3Y-TZP)	Cercon ht (A2)	ZrO_2_ (rest), Y_2_O_3_ (5 wt%), HfO_2_ (3 wt%), Al_2_O_3_, SiO_2_ (<1 wt%)	Dentsply Sirona	18035989	ZR
Lithium disilicate	e-max CAD HT (A2)	SiO_2_ (57.0–80.0 wt%), Li_2_O (11.0–19.0 wt%), K_2_O (<13.0 wt%), Other oxides (<8 wt%)	Ivoclar Vivadent	Y08507	LS
Dental porcelain	Initial MC (E59)	Feldspathic ceramic (N.P.)	GC	1812111	DP
Resin composite	Gradia Forte (CT4)	UDMA (20 wt%), Multifunctional methacrylate (4 wt%), Inorganic fillers (73 wt%), Prepolymerized fillers (3 wt%), Photoinitiators, Stabilizers, Pigments	GC	180591	RC
Bovine tooth	-	-	-	-	BE

3Y-TZP, 3 mol% yttria-stabilized tetragonal zirconia polycrystal; UDMA, urethane dimethacrylate; N.P., not published. * As disclosed by the manufacturers.

**Table 2:** Mean ± standard deviation of Vickers hardness of the specimens.

Experimental Group	ZR	LS	DP	RC	BE
Vickers hardness (Hv)	1287 ± 33 ^a^	604 ± 12 ^b^	492 ± 16 ^c^	124 ± 4 ^d^	303 ± 14 ^e^

One-way ANOVA followed by Tukey’s test. Different lowercase letters are significantly different (*p* < 0.05). Abbreviations of each experimental group are shown in Table 1.

**Table 3:** Mean ± standard deviation of surface roughness (*Sa*) of the abrader specimens.

Experimental Group	ZR	LS	DP	RC	BE
*Sa* (µm)	7.9 ± 2.2 ^a^	8.9 ± 1.9 ^a^	8.1 ± 1.8 ^a^	10.6 ± 3.6 ^a^	6.1 ± 0.5 ^a^

One-way ANOVA followed by Tukey’s test. Same lowercase letters are not significantly different (*p* > 0.05). Abbreviations of each experimental group are shown in Table 1.

**Table 4:** Median of worn depth and width of the substrate specimens.

Experimental Group	ZR	LS	DP	RC	BE
Worn width (mm)	0.61 ^A^	0.81 ^A^	0.93 ^A^	0.49 ^A^	1.67 ^B^
Worn depth (µm)	35.54 ^a^	1.57 ^c^	9.87 ^b^	0.94 ^c^	34.08 ^a^

Kruskal–Wallis test followed by Steel–Dwass post hoc test. Different uppercase and lowercase letters are significantly different (*p* < 0.05). Abbreviations of each experimental group are shown in Table 1.

**Table 5:** Median of worn area and volume of the substrate specimens.

Experimental Group	ZR	LS	DP	RC	BE
Worn area (mm^2^)	2.57 ^A^	4.46 ^A^	4.60 ^A^	2.62 ^A^	10.23 ^B^
Worn volume (mm^3^)	0.07 ^a^	0.03 ^a,b^	0.05 ^a,b^	0.02 ^b^	0.26 ^c^

Kruskal–Wallis test followed by Steel–Dwass post hoc test. Different uppercase and lowercase letters are significantly different (*p* < 0.05). Abbreviations of each experimental group are shown in Table 1.

**Table 6:** Median of worn area of the abrader specimens.

Experimental Group	ZR	LS	DP	RC	BE
Worn area (mm^2^)	0.004 ^a^	1.077 ^b^	1.252 ^b^	0.326 ^c^	3.651 ^d^

Kruskal–Wallis test followed by Steel–Dwass post hoc test. Different lowercase letters are significantly different (*p* < 0.05). Abbreviations of each experimental group are shown in Table 1.

## Data Availability

All data are included in the manuscript.
